# Analytical Model of a Wireless Sensor Network (WSN) Node Operation with a Modified Threshold-Type Energy Saving Mechanism

**DOI:** 10.3390/s19143114

**Published:** 2019-07-14

**Authors:** Wojciech M. Kempa

**Affiliations:** Faculty of Applied Mathematics, Silesian University of Technology, 23 Kaszubska Str., 44-100 Gliwice, Poland; wojciech.kempa@polsl.pl; Tel.: +48-32-237-2864

**Keywords:** energy saving, multiple vacation policy, queue-size distribution, threshold policy, transient state

## Abstract

In this article, a model of the operation of a wireless sensor network (WSN) node with an energy saving mechanism based on a threshold-controlled multiple vacation policy is considered. When the queue of packets directed to the node becomes empty, a multiple vacation period is started during which the receiving/transmitting of packets is blocked. In such a period, successive vacations of a fixed constant duration are taken until a predetermined number of *N* packets accumulated in the queue is detected. Then, at the completion epoch of this vacation, the processing restarts normally. The analytic approach is based on the conception of an embedded Markov chain; integral equations and renewal theory are applied to study the queue-size transient behaviour. The representations for the Laplace transforms of the queue-size distribution at an arbitrary fixed time *t* and on the idle and processing periods are obtained. The compact-form formulae for the distributions of the idle and processing period duration are derived. Numerical examples are attached as well.

## 1. Introduction

The issue of energy saving in wireless sensor networks (WSNs) is crucial from the point of view of network security, including ensuring the reliability of its operation, but also in securing an adequate level of data transmission quality. One of the important tools supporting these processes are the algorithms used for self-organization of sensor networks. The purpose of this type of algorithm, after the initial organization of the network, is primarily to minimize the risk of data loss during transmission, which is associated with the organization of network node structures, and secondly to maximize the battery life of individual sensors (see e.g., [1,2,3]). Another challenge for ensuring the appropriate level of the quality of service (QoS) is the occurrence of temporal death in some sensor nodes (see e.g., [4,5]). There are two different main approaches to solving the problem of energy saving in WSNs. The first one is based on the selection of the shortest route, so uses various-type shortest path algorithms. However, as it turns out, minimizing the costs of energy consumption can lead to an increase in the ratio of lost packets. Hence, another approach chooses not necessarily the shortest route, but one that gives a relatively small ratio of lost packets. As a consequence, the connection may be established using rather peripherally located network nodes, however, they are those for which the data loss ratio is very small (see e.g., [6,7,8,9,10,11] for proposals and discussion on this topic).

In the analysis of the functioning of packet network nodes and in the process of network optimization, queueing theory is often used. The subjects of its research are stochastic characteristics of models in which the individual elements (jobs, packets, items etc.) of the input stream are being processed, but due to insufficient service speed or limitations in the processing process, there occur phenomena like the accumulation of jobs waiting to start the service and sometimes (in the case of finite accumulating buffer capacity) the loss of incoming jobs.

In [12] (compare also [13,14]) the queueing model of the M/M/1-type with a control mechanism based on the *N*-policy is proposed as a tool for reducing power consumption of nodes in wireless sensor networks. A Geo/G/1-type discrete-time model with disasters is considered in [15]. The approach based on Petri nets is proposed in [16] in energy consumption evaluation. A queueing model with priorities is investigated in [17]. Transient (time-dependent, non-stationary) results for queueing systems with multiple (repeated) vacation policy, used as a model of energy saving mechanisms, can be found in [18,19,20,21] (compare also [22,23,24]). In particular, in [18,21] the departure process is investigated in cases of infinite and finite buffer capacities, respectively, while in [19] the queueing delay is investigated (also for the single vacation policy). Similarly, transient analysis of power saving models based on the *N*-policy can be found in [25] (queueing delay) and [26] (departure process).

In the article we study a model of the operation of a WSN node with an energy saving mechanism based on a threshold-controlled multiple vacation policy. When the queue of incoming packets directed to the node empties, a multiple vacation period is initialized, during which the receiving/transmitting of packets is blocked. In such a period, successive vacations of a fixed constant duration are taken until a predetermined number of *N* packets accumulated in the queue is detected. Then, at the completion epoch of this vacation, the processing restarts normally.

We propose the analytic approach based on the conception of an embedded Markov chain, integral equations and renewal theory to investigate the queue-size transient distribution. The explicit compact-form representation for the Laplace transform of the queue-size distribution at arbitrary fixed time *t* and on the idle and processing periods are obtained. The compact-form formulae for the distributions of the idle and processing period duration are derived. A numerical illustration example is attached as well.

The remaining part of the paper is organized as follows. In the next Section 2 we state a detailed mathematical description of the considered queueing system. In Section 3 we analyze the queue-size distribution on the idle period of the system and, moreover, in Section 4 and Section 5 we give explicit results for the probability mass function of the number of packets present in the system at the idle period completion epoch, and the distribution of the idle period duration Laplace transform (LT), respectively. The queue-size distribution on the processing (busy) period is studied in Section 6. The representation for the LT of the distribution of the busy period duration can be found in Section 7. Finally, Section 8 contains the most general result in that the queue-size at an arbitrary epoch is considered.

## 2. Queueing Model Description

In the article we consider an M/G/1-type queueing model with finite buffer capacity and a control (energy saving) mechanism being a mix of the classical multiple vacation policy and the threshold-type policy (*N*-policy). Successive data packets arrive according to a Poisson process with rate λ and are being processed according to a FIFO (First-In-First-Out) service discipline with generally-distributed processing times with a cumulative distribution function (CDF) F(·) and with its Laplace-Stieltjes transform (LST) f(·). The capacity of the accumulating buffer is finite and equals K−1, so the maximum system size is K.

Every time the queue empties an energy saving mode is initialized, during which the processing is completely blocked (idle period). Namely, successive server vacations of constant length *T* are started until the number of accumulated (and waiting for processing) packets equals 1≤N≤K or more, where *N* is fixed. In the occurrence of such a situation, at the moment of this “successful” queue-size measurement the idle period completes and a new processing (busy) period begins immediately.

## 3. Queue-Size on Processing Suspension Period

Let us investigate, firstly, the queue-size behaviour on the first processing suspension period (idle period) $I_{1}$ that starts at time t=0 and consists of a number of independent constant-length vacations being initialized as far as the buffer queue length at the end of one of them will reach at least the predetermined level 1≤N≤K. Evidently, we have

(1) $$\begin{array}{c} P\left\{X\left(t\right)=m,t\in I_{1}\right\}=\sum_{i=1}^{\infty }P\left\{X\left(t\right)=m,t\in I_{1,i}\right\}, \end{array}$$

where $I_{1,i}$ stands for the *i*th successive vacation during the first idle period $I_{1}$.

Let us denote by $1_{A}\left(\cdot \right)$ the characteristic function of the set *A*, i.e.,

(2) $$\begin{array}{c} 1_{A}\left(x\right)\overset{def}{=}\left\{\begin{array}{cc} 1,\; & \mathrm{if}\;x\in A,\\ 0,\; & \mathrm{if}\;x\notin A, \end{array}\right. \end{array}$$

and let $\delta_{i,j}$ stand for the Kronecker delta function.

Note that the following representation holds:

(3) $$\begin{array}{c} P\left\{X\left(t\right)=m,t\in I_{1,i}\right\}=1_{\left[(i-1)T,iT\right)}\left(t\right)\{1_{\left\{0,\ldots ,N-1\right\}}\left(m\right)\sum_{k=0}^{m}\frac{{\left[\lambda \left(i-1\right)T\right]}^{k}}{k!}e^{-\lambda (i-1)T}\\ \cdot \frac{{\left[\lambda \left(t-\left(i-1\right)T\right)\right]}^{m-k}}{(m-k)!}e^{-\lambda [t-(i-1)T]}\\ +1_{\left\{N,\ldots ,K-1\right\}}\left(m\right)\sum_{k=0}^{N-1}\frac{{\left[\lambda \left(i-1\right)T\right]}^{k}}{k!}e^{-\lambda (i-1)T}\frac{{\left[\lambda \left(t-\left(i-1\right)T\right)\right]}^{m-k}}{(m-k)!}e^{-\lambda [t-(i-1)T]}\\ +\delta_{m,K}\sum_{k=0}^{N-1}\frac{{\left[\lambda \left(i-1\right)T\right]}^{k}}{k!}e^{-\lambda (i-1)T}\sum_{j=K-k}^{\infty }\frac{{\left[\lambda \left(t-\left(i-1\right)T\right)\right]}^{j}}{j!}e^{-\lambda [t-(i-1)T]}\}. \end{array}$$

Let us briefly comment on (3). Indeed, if $t\in I_{1,i}$ then t∈[(i−1)T,iT). The first summand on the right side of (3) refers to the situation in which the threshold level *N* is not reached before time t. In the second summand the number of packets accumulated in the buffer queue reaches at least *N* at time *t* but the buffer is still not saturated (clearly, up to time (i−1)T the number of arrivals must equal at most N−1 in order to initialize the *i*th vacation). Finally, the last summand on the right side of (3) describes the situation, in that the buffer is saturated at time t.

Before we will state the representation for the LT of $P\{X\left(t\right)=m,t\in I_{1,i}\}$, let us observe that for s>0 we have

(4) $$\begin{array}{c} \int_{(i-1)T}^{iT}e^{-(s+\lambda )t}\frac{{\left[\lambda \left(t-\left(i-1\right)T\right)\right]}^{j}}{j!}dt\\ =\int_{0}^{T}e^{-(s+\lambda )[u+(i-1)T]}\frac{{\left(\lambda u\right)}^{j}}{j!}du=e^{-(s+\lambda )(i-1)T}\frac{\lambda^{j}}{{\left(s+\lambda \right)}^{j+1}}E_{s+\lambda }\left(j,T\right), \end{array}$$

where $E_{s+\lambda }\left(j,\cdot \right)$ denotes the CDF of the *j*-Erlang distribution with parameter s+λ.

Referring to (2)–(4), we get now for s>0

(5) $$\begin{array}{c} p_{I}\left(s,m\right)\overset{def}{=}\int_{0}^{\infty }e^{-st}P\left\{X\left(t\right)=m,t\in I_{1}\right\}dt=\sum_{i=1}^{\infty }e^{-(s+\lambda )(i-1)T}\{1_{\left\{0,\ldots ,N-1\right\}}\left(m\right)\\ \cdot \sum_{k=0}^{m}\frac{\lambda^{m-k}}{{\left(s+\lambda \right)}^{m-k+1}}\cdot \frac{{\left[\lambda \left(i-1\right)T\right]}^{k}}{k!}E_{s+\lambda }\left(m-k,T\right)+\sum_{k=0}^{N-1}\frac{{\left[\lambda \left(i-1\right)T\right]}^{k}}{k!}\\ \cdot \left[1_{\left\{N,\ldots ,K-1\right\}}\left(m\right)\frac{\lambda^{m-k}}{{\left(s+\lambda \right)}^{m-k+1}}E_{s+\lambda }\left(m-k,T\right)+\delta_{m,K}\sum_{j=K-k}^{\infty }\frac{\lambda^{j}}{{\left(s+\lambda \right)}^{j+1}}E_{s+\lambda }\left(j,T\right)\right]\}. \end{array}$$

## 4. Queue Measurement at the Idle Period Completion Epoch

In this section we are interested in the probability distribution of the queue state $X_{I_{r}}=X_{I}$ at the completion epoch of the arbitrary processing suspension period (idle period) $I_{r}$.

Denoting

(6) $$\begin{array}{c} a_{n}\overset{def}{=}P\left\{X_{I}=n\right\},\;n\in \left\{N,\ldots ,K\right\}, \end{array}$$

observe that the following equality is true:

(7) $$\begin{array}{c} a_{n}=\sum_{i=1}^{\infty }P\left\{X\left(iT\right)=n,\;\mathrm{idle}\;\mathrm{period}\;\mathrm{contains}\;\mathrm{exactly}\;i\;\mathrm{single}\;\mathrm{vacations}\right\}\\ =\sum_{i=1}^{\infty }(1_{\left\{N,\ldots ,K-1\right\}}\left(n\right)\sum_{k=0}^{N-1}\frac{{\left[\lambda \left(i-1\right)T\right]}^{k}}{k!}e^{-\lambda (i-1)T}\cdot \frac{{\left(\lambda T\right)}^{n-k}}{(n-k)!}e^{-\lambda T}\\ +\delta_{n,K}\sum_{k=0}^{N-1}\frac{{\left[\lambda \left(i-1\right)T\right]}^{k}}{k!}e^{-\lambda (i-1)T}\sum_{j=K-k}^{\infty }\frac{{\left(\lambda T\right)}^{j}}{j!}e^{-\lambda T}). \end{array}$$

Indeed, the first summand on the right side of (7) refers to the situation where the buffer is not fulfilled before the endpoint of the idle period, while for the second one the processing starts after the idle period with exactly *K* packets present.

## 5. Idle Period Duration

In this section, without loss of generality, we identify the *r*th idle period $I_{r}$ with its duration. Obviously, we have

(8) $$\begin{array}{c} P\left\{I_{r}\geq iT\right\}=\sum_{k=0}^{N-1}\frac{{\left[\lambda \left(i-1\right)T\right]}^{k}}{k!}e^{-\lambda (i-1)T},\;r=1,2,\ldots , \end{array}$$

due to the fact that the *i*th vacation during the idle period is being initialized if and only if the number of accumulated packets at time (i−1)T does not exceed N−1.

From (8) we obtain immediately

(9) $$\begin{array}{c} P\left\{I_{r}=iT\right\}=P\left\{I_{r}\geq iT\right\}-P\left\{I_{r}\geq \left(i+1\right)T\right\}\\ =\sum_{k=0}^{N-1}\left(\frac{{\left[\lambda \left(i-1\right)T\right]}^{k}}{k!}e^{-\lambda (i-1)T}-\frac{{\left(\lambda iT\right)}^{k}}{k!}e^{-\lambda iT}\right). \end{array}$$

Introducing the LST $f_{I}\left(\cdot \right)$ of the CDF of the arbitrary idle period duration, we get

(10) $$\begin{array}{c} f_{I}\left(s\right)\overset{def}{=}\sum_{i=0}^{\infty }e^{-siT}\left(P\left\{I_{r}=\left(i+1\right)T\right\}-P\left\{I_{r}=iT\right\}\right)=\sum_{i=0}^{\infty }e^{-siT}\\ \cdot \sum_{k=0}^{N-1}\left(\frac{2{\left(\lambda iT\right)}^{k}}{k!}e^{-\lambda iT}-\frac{{\left[\lambda \left(i+1\right)T\right]}^{k}}{k!}e^{-\lambda (i+1)T}-\frac{{\left[\lambda \left(i-1\right)T\right]}^{k}}{k!}e^{-\lambda (i-1)T}\right). \end{array}$$

## 6. Queue-Size on Processing Period

We investigate now the case of the queue-size distribution on a single processing period. Obviously, the transient behavior of this stochastic characteristic depends on the system state *n* at the completion epoch of the preceding idle period, where n∈{N,…,K}. Without loss of generality, let us assume that the processing period starts at time t=0 and, temporarily, that at the starting moment the number of packets accumulated in the buffer can take on any value from {1,…,K}. To receive a representation for the LT of the conditional queue-size distribution on a processing period, we will use the method of potential proposed by Korolyuk in [27]. Let us denote by X(t) the number of packets present in the system exactly at time t.

Introduce the following notations:

(11) $$\begin{array}{c} P_{n}^{\left(P\right)}\left(t,m\right)\overset{def}{=}P\left\{X\left(t\right)=m,t\;\mathrm{is}\;\mathrm{in}\;a\;\mathrm{processing}\;\mathrm{period}\;|\;X\left(0\right)=n\right\}, \end{array}$$

where m,n∈{1,…,K} and t>0, and

(12) $$\begin{array}{c} p_{n}^{\left(P\right)}\left(s,m\right)\overset{def}{=}\int_{0}^{\infty }e^{-st}P_{n}^{\left(P\right)}\left(t,m\right)dt,\;s>0. \end{array}$$

It is well known that, due to the memoryless property of the exponential distribution of interarrival times, the sequence $X(t_{i})$, i=1,2,…, where $t_{i}$ stands for the *i*th processing completion epoch after the starting moment, is an embedded Markov chain for the process {X(t),t≥0}. Thus, utilizing the formula of total probability with respect to the first departure moment after t=0, we obtain for n=1

(13) $$\begin{array}{c} P_{1}^{\left(P\right)}\left(t,m\right)=\sum_{i=1}^{K-2}\int_{0}^{t}\frac{{\left(\lambda x\right)}^{i}}{i!}e^{-\lambda x}P_{i}^{\left(P\right)}\left(t-x,m\right)dF\left(x\right)\\ +\sum_{i=K-1}^{\infty }\int_{0}^{t}\frac{{\left(\lambda x\right)}^{i}}{i!}e^{-\lambda x}P_{K-1}^{\left(P\right)}\left(t-x,m\right)dF\left(x\right)\\ +\left[1-F\left(t\right)\right]e^{-\lambda t}\left(1_{\left\{1,\ldots ,K-1\right\}}\left(m\right)\frac{{\left(\lambda t\right)}^{m-1}}{(m-1)!}+\delta_{m,K}\sum_{i=K-1}^{\infty }\frac{{\left(\lambda t\right)}^{i}}{i!}\right). \end{array}$$

Similarly, for 2≤n≤K, we have

(14) $$\begin{array}{c} P_{n}^{\left(P\right)}\left(t,m\right)=\sum_{i=0}^{K-n-1}\int_{0}^{t}\frac{{\left(\lambda x\right)}^{i}}{i!}e^{-\lambda x}P_{n+i-1}^{\left(P\right)}\left(t-x,m\right)dF\left(x\right)\\ +\sum_{i=K-n}^{\infty }\int_{0}^{t}\frac{{\left(\lambda x\right)}^{i}}{i!}e^{-\lambda x}P_{K-1}^{\left(P\right)}\left(t-x,m\right)dF\left(x\right)\\ +\left[1-F\left(t\right)\right]e^{-\lambda t}\left(1_{\left\{n,\ldots ,K-1\right\}}\left(m\right)\frac{{\left(\lambda t\right)}^{m-n}}{(m-n)!}+\delta_{m,K}\sum_{i=K-n}^{\infty }\frac{{\left(\lambda t\right)}^{i}}{i!}\right). \end{array}$$

Observe that the first summand on the right side of (13) and (14) describes the case in that the accumulating buffer does not become fulfilled before the first departure occurring precisely at time x<t, while the second one refers to the situation in which the system is saturated before x. The case of the first processing finishing after *t* is presented in the last summand on the right side of (13) and (14). Let us note that the first sum in (14) is taken from 0 while in (13) from 1. Indeed, if the system contains exactly one packet at the starting moment then no departure occurs before t, otherwise the processing (busy) period will end before t.

Let us define for s>0 the following functional sequences:

(15) $$\begin{array}{c} \gamma_{n}\left(s\right)\overset{def}{=}\int_{0}^{\infty }e^{-(\lambda +s)t}\frac{{\left(\lambda t\right)}^{n}}{n!}dF\left(t\right) \end{array}$$

and

(16) $$\begin{array}{c} \eta_{n}\left(s,m\right)\overset{def}{=}\int_{0}^{\infty }e^{-(\lambda +s)t}\left[1-F\left(t\right)\right]\left[1_{\left\{n,\ldots ,K-1\right\}}\left(m\right)\frac{{\left(\lambda t\right)}^{m-n}}{(m-n)!}+\delta_{m,K}\sum_{i=K-n}^{\infty }\frac{{\left(\lambda t\right)}^{i}}{i!}\right]dt. \end{array}$$

Implementing (15) and (16) into (13) and (14) gives

(17) $$\begin{array}{c} p_{1}^{\left(P\right)}\left(s,m\right)=\sum_{i=1}^{K-2}\gamma_{i}\left(s\right)p_{i}^{\left(P\right)}\left(s,m\right)+p_{K-1}^{\left(P\right)}\left(s,m\right)\sum_{i=K-1}^{\infty }\gamma_{i}\left(s\right)+\eta_{1}\left(s,m\right) \end{array}$$

and

(18) $$\begin{array}{c} p_{n}^{\left(P\right)}\left(s,m\right)=\sum_{i=0}^{K-n-1}\gamma_{i}\left(s\right)p_{n+i-1}^{\left(P\right)}\left(s,m\right)+p_{K-1}^{\left(P\right)}\left(s,m\right)\sum_{i=K-n}^{\infty }\gamma_{i}\left(s\right)+\eta_{n}\left(s,m\right), \end{array}$$

where 2≤n≤K.

Let us rewrite equations of the system (17) and (18) in a another form. Indeed, substituting ${\tilde{p}}_{n}^{\left(P\right)}\left(s,m\right)\overset{def}{=}p_{K-n}^{\left(P\right)}\left(s,m\right),$ where 0≤n≤K−1, we transform them as follows:

(19) $$\begin{array}{c} {\tilde{p}}_{K-1}^{\left(P\right)}\left(s,m\right)=\sum_{i=1}^{K-2}\gamma_{i}\left(s\right){\tilde{p}}_{K-i}^{\left(P\right)}\left(s,m\right)+{\tilde{p}}_{1}^{\left(P\right)}\left(s,m\right)\sum_{i=K-1}^{\infty }\gamma_{i}\left(s\right)+\eta_{1}\left(s,m\right), \end{array}$$

(20) $$\begin{array}{c} \sum_{k=-1}^{n}\gamma_{k+1}\left(s\right){\tilde{p}}_{n-k}^{\left(P\right)}\left(s,m\right)-{\tilde{p}}_{n}^{\left(P\right)}\left(s,m\right)=\omega_{n}\left(s,m\right), \end{array}$$

where 0≤n≤K−2, and

(21) $$\begin{array}{c} \omega_{n}\left(s,m\right)\overset{def}{=}\gamma_{n+1}\left(s\right){\tilde{p}}_{0}^{\left(P\right)}\left(s,m\right)-{\tilde{p}}_{1}^{\left(P\right)}\left(s,m\right)\sum_{i=n+1}^{\infty }\gamma_{i}\left(s\right)-\eta_{K-n}\left(s,m\right). \end{array}$$

Each solution of the linear infinite-size system with known coefficients $a_{k}$’s and $b_{k}$’s

(22) $$\begin{array}{c} \sum_{k=-1}^{n}a_{k+1}x_{n-k}-x_{n}=b_{n},\;n\geq 0, \end{array}$$

can be written in the following form (see [27,28]):

(23) $$\begin{array}{c} x_{n}=cr_{n+1}+\sum_{i=0}^{n}r_{n-i}b_{i}, \end{array}$$

where *c* is certain constant and the sequence $\left(r_{n}\right)$ is defined by the following recursive relationship:

(24) $$\begin{array}{c} r_{n}=\left\{\begin{array}{cc} 0, & \mathrm{for}\;n=0,\\ 1/a_{0}, & \mathrm{for}\;n=1,\\ \left(1/a_{0}\right)\left(r_{n-1}-\sum_{i=0}^{n-1}a_{i+1}r_{n-1-i}\right), & \mathrm{for}\;n\geq 2. \end{array}\right. \end{array}$$

At this stage let us make some essential observations. Firstly, the systems (20) and (22) are very similar, however in the former, functional sequences occur. Secondly, the number of equations in (20) is finite, while in (22) it is infinite. As it turns out, the representation (23) can be used to find the solution of the system (19) and (20) and, moreover, the fact that the number of equations in (20) is finite allows for finding an explicit representation for c.

So, we can write (compare (23) and (24))

(25) $$\begin{array}{c} {\tilde{p}}_{n}^{\left(P\right)}\left(s,m\right)=c\left(s,m\right)r_{n+1}\left(s\right)+\sum_{i=0}^{n}r_{n-i}\left(s\right)\omega_{i}\left(s,m\right), \end{array}$$

where c(s,m) does not depend on the index *n* and the functional sequence $\left(r_{n}\left(s\right)\right)$ is defined via $\left(\gamma_{n}\left(s\right)\right)$ in the following way:

(26) $$\begin{array}{c} r_{n}\left(s\right)=\left\{\begin{array}{cc} 0, & \mathrm{for}\;n=0,\\ 1/\gamma_{0}\left(s\right), & \mathrm{for}\;n=1,\\ \left(1/\gamma_{0}\left(s\right)\right)\left[r_{n-1}-\sum_{i=0}^{n-1}\gamma_{i+1}\left(s\right)r_{n-1-i}\left(s\right)\right], & \mathrm{for}\;n\geq 2. \end{array}\right. \end{array}$$

Note that to represent ${\tilde{p}}_{n}^{\left(P\right)}\left(s,m\right)$ explicitly we need to find the formulae for ${\tilde{p}}_{0}^{\left(P\right)}\left(s,m\right)$ and ${\tilde{p}}_{1}^{\left(P\right)}\left(s,m\right)$ occurring in the definition (21) of $\omega_{n}\left(s,m\right)$.

Let us start with substituting n=0 into (25). We obtain

(27) $$\begin{array}{c} c\left(s,m\right)=\gamma_{0}\left(s\right){\tilde{p}}_{0}^{\left(P\right)}\left(s,m\right). \end{array}$$

Writing (20) for n=0 leads to

(28) $$\begin{array}{c} {\tilde{p}}_{1}^{\left(P\right)}\left(s,m\right)={\left[\gamma_{0}\left(s\right)\right]}^{-1}[\omega_{0}\left(s,m\right)+{\tilde{p}}_{0}^{\left(P\right)}\left(s,m\right)\left(1-\gamma_{1}\left(s\right)\right)] \end{array}$$

and hence, referring to (21), we get

(29) $$\begin{array}{c} {\tilde{p}}_{1}^{\left(P\right)}\left(s,m\right)=\frac{{\tilde{p}}_{0}^{\left(P\right)}\left(s,m\right)-\eta_{K}\left(s,m\right)}{f(s)}. \end{array}$$

As a consequence, implementing representations (27) and (29) in the formula (25), we have

(30) $$\begin{array}{c} {\tilde{p}}_{n}^{\left(P\right)}\left(s,m\right)=g_{n}\left(s\right){\tilde{p}}_{0}^{\left(P\right)}\left(s,m\right)+h_{n}\left(s,m\right), \end{array}$$

where n≥0 and

(31) $$\begin{array}{c} g_{n}\left(s\right)\overset{def}{=}\gamma_{0}\left(s\right)r_{n+1}\left(s\right)+\sum_{i=0}^{n}r_{n-i}\left(s\right)\{\gamma_{i+1}\left(s\right)-{\left[f\left(s\right)\right]}^{-1}\sum_{j=i+1}^{\infty }\gamma_{j}\left(s\right)\} \end{array}$$

and

(32) $$\begin{array}{c} h_{n}\left(s,m\right)\overset{def}{=}\sum_{i=0}^{n}r_{n-i}\left(s\right)\{{\left[f\left(s\right)\right]}^{-1}\eta_{K}\left(s,m\right)\sum_{j=i+1}^{\infty }\gamma_{j}\left(s\right)-\eta_{K-i}\left(s,m\right)\}. \end{array}$$

Applying now the representation (30) in the Equation (19), we can easily eliminate ${\tilde{p}}_{0}^{\left(P\right)}\left(s,m\right)$ in the form

(33) $$\begin{array}{c} {\tilde{p}}_{0}^{\left(P\right)}\left(s,m\right)=U\left(s,m\right)V\left(s\right), \end{array}$$

where

(34) $$\begin{array}{c} U\left(s,m\right)\overset{def}{=}\sum_{i=1}^{K-2}\gamma_{i}\left(s\right)h_{K-i}\left(s,m\right)-{\left[f\left(s\right)\right]}^{-1}\eta_{K}\left(s,m\right)\sum_{i=K-1}^{\infty }\gamma_{i}\left(s\right)+\eta_{1}\left(s,m\right)-h_{K-1}\left(s,m\right), \end{array}$$

(35) $$\begin{array}{c} V\left(s\right)\overset{def}{=}{\left\{h_{K-1}\left(s\right)-\sum_{i=1}^{K-2}\gamma_{i}\left(s\right)g_{K-i}\left(s\right)-{\left[f\left(s\right)\right]}^{-1}\sum_{i=K-1}^{\infty }\gamma_{i}\left(s\right)\right\}}^{-1}. \end{array}$$

Finally, returning to $p_{n}^{\left(P\right)}\left(s,m\right)$ (instead of ${\tilde{p}}_{n}^{\left(P\right)}\left(s,m\right)$) and referring to (21), (25), (27), (29) and (33), we can formulate the following result:

(36) $$\begin{array}{cc} p_{n}^{\left(P\right)}\left(s,m\right)= & \int_{0}^{\infty }e^{-st}P_{n}^{\left(P\right)}\left(t,m\right)dt=\left\{\gamma_{0}\left(s\right)r_{K-n+1}\left(s\right)+\sum_{i=0}^{K-n}r_{K-n-i}\left(s\right)\left[\gamma_{i+1}\left(s\right)-{\left(f\left(s\right)\right)}^{-1}\sum_{j=i+1}^{\infty }\gamma_{j}\left(s\right)\right]\right\}\\ & \cdot U\left(s,m\right)V\left(s\right)+\sum_{i=0}^{K-n}r_{K-n-i}\left(s\right)\left[{\left(f\left(s\right)\right)}^{-1}\eta_{K}\left(s,m\right)\sum_{j=i+1}^{\infty }\gamma_{j}\left(s\right)-\eta_{K-i}\left(s,m\right)\right], \end{array}$$

where s>0 and the formulae for $\gamma_{k}\left(s\right)$, $\eta_{k}\left(s,m\right)$, $r_{k}\left(s\right)$, U(s,m) and V(s) are given in (15), (16), (26), (34) and (35), respectively.

## 7. Processing Period Duration

In this section we establish the formula for the LST of the first busy (processing) period duration, conditioned by the number of packets accumulated in the buffer at the starting epoch. The appropriate result we will write in the most general case, namely assuming that the processing period may start with n∈{1,…,K} packets present (not necessarily with at least *N* packets present). Suppose temporarily that the first busy period starts at t=0.

Introduce the following notations:

(37) $$\begin{array}{c} F_{BP,n}\left(x\right)\overset{def}{=}P\left\{\mathrm{first}\;\mathrm{busy}\;\mathrm{period}\;\mathrm{duration}\;<x\;|\;\mathrm{busy}\;\mathrm{period}\;\mathrm{starts}\;\mathrm{with}\;n\;\mathrm{packets}\;\mathrm{present}\right\} \end{array}$$

and

(38) $$\begin{array}{c} f_{BP,n}\left(s\right)\overset{def}{=}\int_{0}^{\infty }e^{-sx}dF_{BP,n}\left(x\right),\;s>0. \end{array}$$

It is easy to note that for $f_{BP,n}\left(s\right)$, n∈{1,…,K}, the following system of equations is true (compare (17) and (18)):

(39) $$\begin{array}{c} f_{BP,1}\left(s\right)=\sum_{i=1}^{K-2}\gamma_{i}\left(s\right)f_{BP,i}\left(s\right)+f_{BP,K-1}\left(s\right)\sum_{i=K-1}^{\infty }\gamma_{i}\left(s\right)+f\left(\lambda +s\right), \end{array}$$

(40) $$\begin{array}{c} f_{BP,n}\left(s\right)=\sum_{i=0}^{K-n-1}\gamma_{i}\left(s\right)f_{BP,n+i-1}\left(s\right)+f_{BP,K-1}\left(s\right)\sum_{i=K-n}^{\infty }\gamma_{i}\left(s\right), \end{array}$$

where 2≤n≤K.

Let us observe that if we define

(41) $$\begin{array}{c} {\tilde{\eta }}_{n}\left(s,m\right)\overset{def}{=}\delta_{n,1}f\left(\lambda +s\right), \end{array}$$

then we can obtain the explicit-form representation for $f_{BP,n}\left(s\right)$ utilizing the Formula (36). Indeed, we obtain

(42) $$\begin{array}{cc} f_{BP,n}\left(s,m\right)= & \left\{\gamma_{0}\left(s\right)r_{K-n+1}\left(s\right)+\sum_{i=0}^{K-n}r_{K-n-i}\left(s\right)\left[\gamma_{i+1}\left(s\right)-{\left(f\left(s\right)\right)}^{-1}\sum_{j=i+1}^{\infty }\gamma_{j}\left(s\right)\right]\right\}\\ & \cdot \tilde{U}\left(s,m\right)V\left(s\right)+\sum_{i=0}^{K-n}r_{K-n-i}\left(s\right)\left[{\left(f\left(s\right)\right)}^{-1}{\tilde{\eta }}_{K}\left(s,m\right)\sum_{j=i+1}^{\infty }\gamma_{j}\left(s\right)-{\tilde{\eta }}_{K-i}\left(s,m\right)\right], \end{array}$$

where s>0 and now

(43) $$\begin{array}{c} \tilde{U}\left(s,m\right)\overset{def}{=}\sum_{i=1}^{K-2}\gamma_{i}\left(s\right){\tilde{h}}_{K-i}\left(s,m\right)-{\left[f\left(s\right)\right]}^{-1}{\tilde{\eta }}_{K}\left(s,m\right)\sum_{i=K-1}^{\infty }\gamma_{i}\left(s\right)+{\tilde{\eta }}_{1}\left(s,m\right)-{\tilde{h}}_{K-1}\left(s,m\right) \end{array}$$

and

(44) $$\begin{array}{c} {\tilde{h}}_{n}\left(s,m\right)\overset{def}{=}\sum_{i=0}^{n}r_{n-i}\left(s\right)\left\{{\left[f\left(s\right)\right]}^{-1}{\tilde{\eta }}_{K}\left(s,m\right)\sum_{j=i+1}^{\infty }\gamma_{j}\left(s\right)-{\tilde{\eta }}_{K-i}\left(s,m\right)\right\}. \end{array}$$

## 8. Queue-Size Distribution at an Arbitrary Time

Consider now the transient queue-size distribution at arbitrary time t. Assume that the system starts the operation at time t=0 with the first idle period. The evolution of the considered queueing system can be observed on successive idle periods $I_{1},I_{2},\ldots$ interspersed with busy periods $BP_{1},BP_{2},\ldots$. The formula of total probability gives

(45) $$\begin{array}{c} P\left\{X\left(t\right)=m\right\}=\sum_{i=1}^{\infty }\left(P\left\{X\left(t\right)=m,\;t\in I_{i}\right\}+P\left\{X\left(t\right)=m,\;t\in BP_{i}\right\}\right). \end{array}$$

Due to the memoryless property of the exponential distribution of interarrival times, the following representation is true:

(46) $$\begin{array}{cc} P\{X\left(t\right)=m,\;t\in I_{i}\}= & \sum_{l_{1},\ldots ,l_{i-1}\in \left\{N,\ldots ,K\right\}}\prod_{j=1}^{i-1}a_{l_{j}}\int_{0}^{t}P\left\{X\left(t-y\right)=m,\;t-y\in I_{1}\right\}\\ & \cdot d\left(F_{I}^{(i-1)i*}*F_{BP,a_{l_{1}}}*\ldots *F_{BP,a_{l_{i-1}}}\right)\left(y\right), \end{array}$$

where $F_{I}\left(\cdot \right)$ stands for the CDF of the idle period duration (all idle periods are independent and identically distributed) and * denotes the Stieltjes convolution. The sum on the right side of (46) is taken over all possible values of $l_{1},\ldots ,l_{i-1}\in \left\{N,\ldots ,K\right\}.$

Quite similarly we obtain

(47) $$\begin{array}{c} P\{X\left(t\right)=m,\;t\in BP_{i}\}\\ =\sum_{l_{1},\ldots ,l_{i}\in \left\{N,\ldots ,K\right\}}\prod_{j=1}^{i-1}a_{l_{j}}\int_{0}^{t}P\left\{X\left(t-y\right)=m,\;t-y\in BP_{1}\;|\;BP_{1}\;\mathrm{starts}\;\mathrm{with}\;l_{i}\;\mathrm{packets}\;\mathrm{present}\right\}\\ \cdot d\left(F_{I}^{i*}*F_{BP,a_{l_{1}}}*\ldots *F_{BP,a_{l_{i-1}}}\right)\left(y\right). \end{array}$$

Taking LTs of both sides in (46) and (47) and referring to (45), we obtain the following representation for the LT of the queue-size distribution in the considered queueing model with a threshold-controlled multiple vacation policy:

(48) $$\begin{array}{cc} \int_{0}^{\infty }e^{-st}P\left\{X\left(t\right)=m\right\}dt= & \sum_{i=1}^{\infty }\{p_{I}\left(s,m\right){\left[f_{I}\left(s\right)\right]}^{i-1}\sum_{l_{1},\ldots ,l_{i-1}\in \left\{N,\ldots ,K\right\}}\prod_{j=1}^{i-1}a_{l_{j}}f_{BP,a_{l_{j}}}\left(s\right)\\ & +\sum_{l_{1},\ldots ,l_{i}\in \left\{N,\ldots ,K\right\}}a_{l_{i}}p_{a_{l_{i}}}^{\left(P\right)}\left(s,m\right)\prod_{j=1}^{i-1}a_{l_{j}}f_{BP,a_{l_{j}}}\left(s\right)\}, \end{array}$$

where s>0.

## 9. Numerical Study

In this section we present some numerical examples illustrating theoretical results obtained in previous sections. Let us investigate the operation of the system on the first idle period. Assume that the intensity of Poisson arrivals equals 1.1 items per time unit (mean interarrival times are equal to 0.909) and that the processing times are distributed according to a mixture of two exponential distributions, namely the probability density function $f_{S}\left(\cdot \right)$ of the service time has the following form:

(49) $$\begin{array}{c} f_{S}\left(t\right)=0.25\cdot 2\exp\left(-2t\right)+0.75\cdot \exp\left(-t\right),\;t>0. \end{array}$$

**Figure 1:**
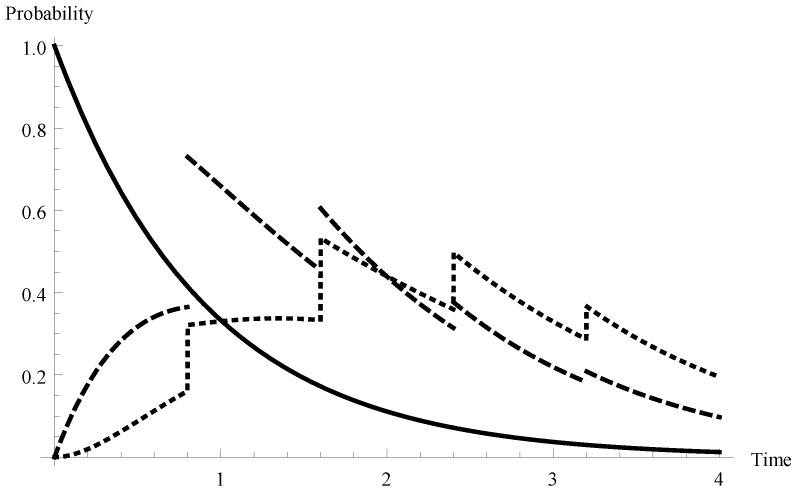
Transient probabilities $P\{X\left(t\right)=m,t\in I_{1}\}$ for λ=1.1 and for m=0,1,2.

Hence the mean service time of a single packet equals 0.875 and, in consequence, the offered load for the system is ϱ=0.963. Let us fix K=8, N=3 and T=0.8. In Figure 1 the queue-size distributions $P\{X\left(t\right)=m,t\in I_{1}\}$ for m=0 (solid line), m=1 (dashed line) and 2 (dotted line) are visualized. As one can observe, for m>0 the graphs have characteristic shapes in which the character of the function changes every 0.8 time unit. Obviously, it is connected with the fixed duration of a single server vacation equal to 0.8.

**Figure 2:**
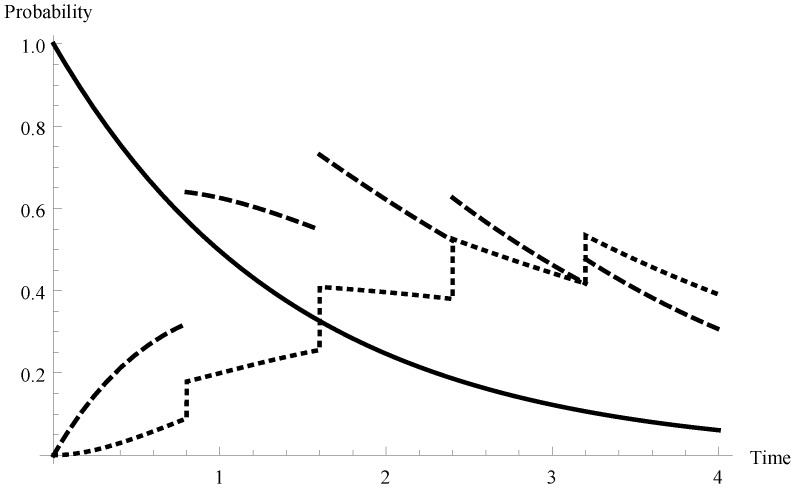
Transient probabilities $P\{X\left(t\right)=m,t\in I_{1}\}$ for λ=0.7 and for m=0,1,2.

**Figure 3:**
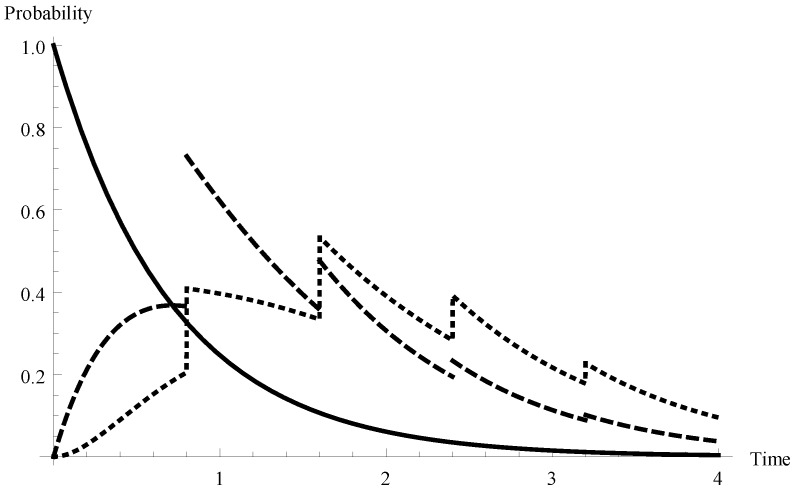
Transient probabilities $P\{X\left(t\right)=m,t\in I_{1}\}$ for λ=1.4 and for m=0,1,2.

In Figure 2 and Figure 3 the appropriate results are presented for two other arrival intensities. In Figure 2 the case of a relatively low arrival intensity λ=0.7 (mean interarrival time equal to 1.429 and the offered load ϱ=0.612) is shown. Similarly, the case of the overloaded system in that λ=1.4 (mean interarrival time equal to 0.714 and the offered load ϱ=1.225) is visualized in Figure 3.

**Figure 4:**
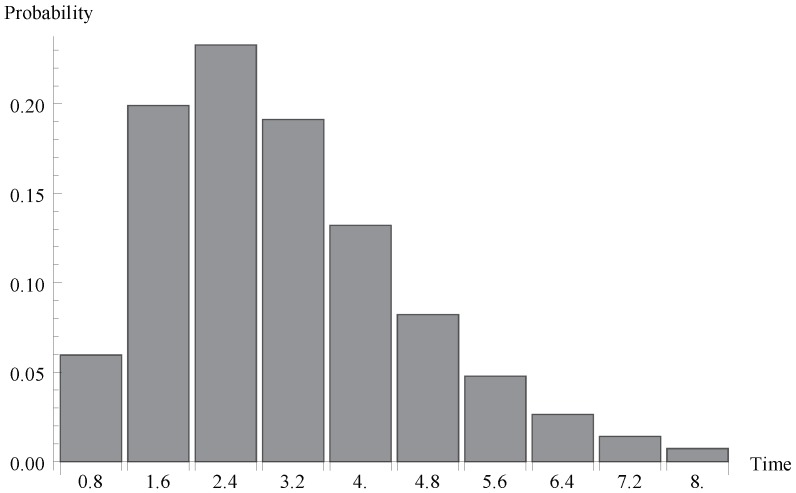
Distribution of idle period duration for λ=1.1.

**Figure 5:**
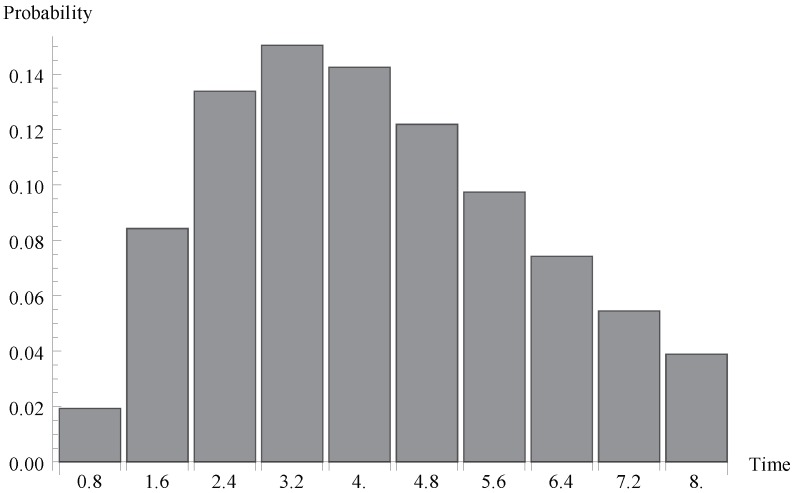
Distribution of idle period duration for λ=0.7.

**Figure 6:**
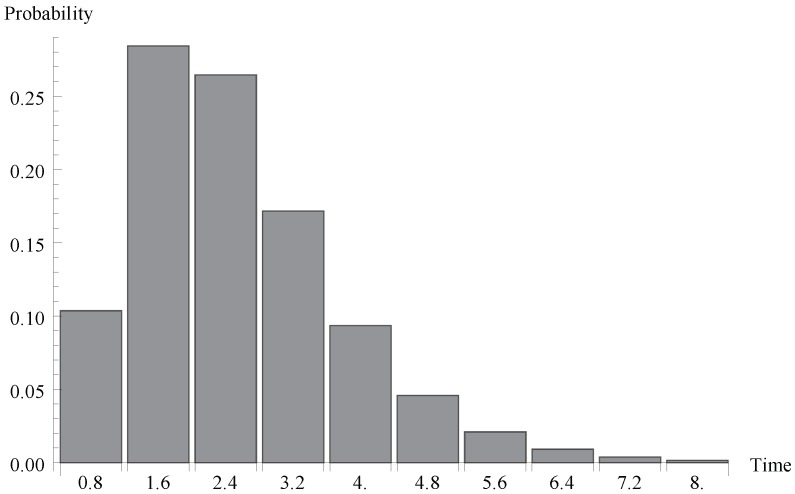
Distribution of idle period duration for λ=1.4.

We investigate now the behaviour of the probability distribution of the idle period duration. Evidently, this time is a certain multiple of the single vacation duration T. In Figure 4, Figure 5 and Figure 6, respectively, the cases of λ=1.1, 0.7 and 1.4 are presented. As it can be observed, the greater arrival intensity, the greater probability that the idle period will finish after one server vacation only (at time T=0.8). Moreover, let us note that (except for the case of one single vacation case) the maximum of the distribution moves to the right with the decrease of λ (or with the decrease of the offered load).

**Figure 7:**
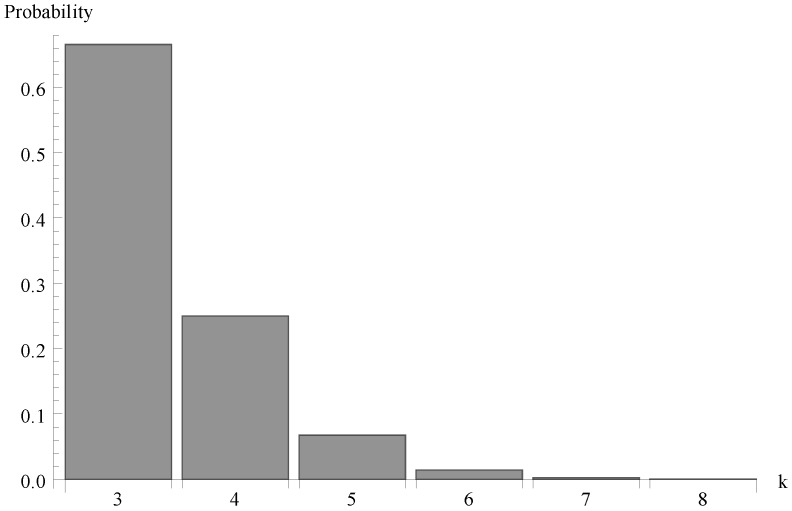
Number of packets of at the completion epoch of idle period for λ=1.1.

**Figure 8:**
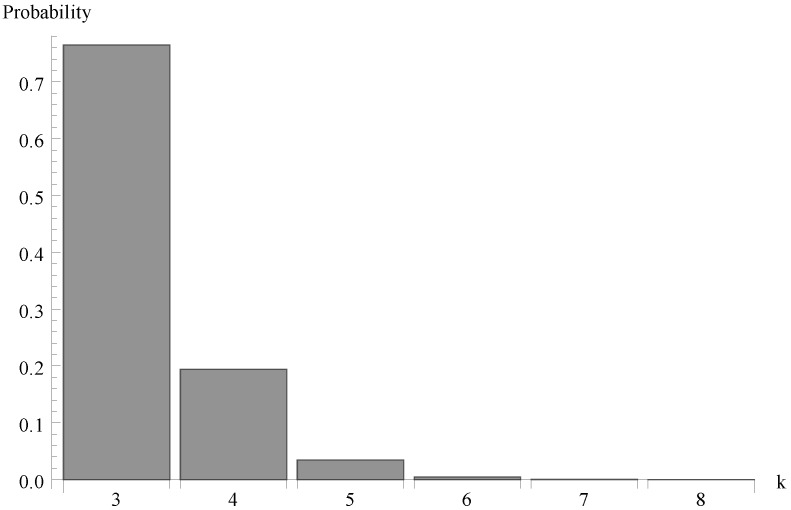
Number of packets of at the completion epoch of idle period for λ=0.7.

**Figure 9:**
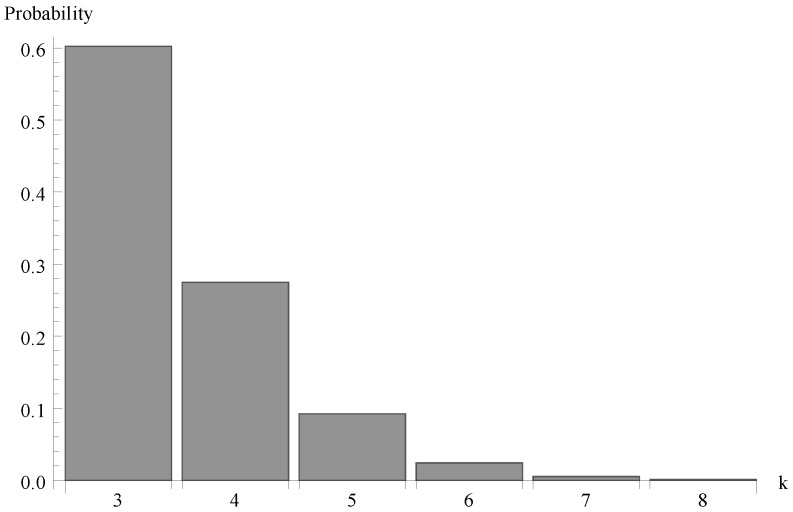
Number of packets of at the completion epoch of idle period for λ=1.4.

Finally, in Figure 7, Figure 8 and Figure 9 the probability distributions of the number of packets present in the system at the completion epoch of the idle period are shown for the same three values of the arrival intensity, so for the offered traffic load ϱ=0.963, 0.612 and 1.225.

## 10. Conclusions

In the paper a queueing system with a threshold-type multiple vacation policy is considered as a model of the operation of a WSN node with an energy saving mechanism. The idea of the algorithm is that the node radio transmitter/receiver goes to sleep mode every time the queue of packets directed to the node becomes empty. Being in this mode it takes successive independent vacations of deterministic duration until the predetermined number of packets present is detected. The time-dependent queue-size distribution is studied, applying the analytic approach based on the idea of the embedded Markov chain, integral equations and renewal theory. The compact-form formulae for the Laplace transforms of the queue-size distribution at an arbitrary fixed time *t*, and on the idle and processing periods are obtained. Moreover, the compact-form formulae for the distributions of the idle and processing period duration are derived. Numerical examples illustrating the theoretical results are obtained.
